# Mesenchymal Stem Cells for the Treatment of Spinal Arthrodesis: From Preclinical Research to Clinical Scenario

**DOI:** 10.1155/2017/3537094

**Published:** 2017-02-13

**Authors:** F. Salamanna, M. Sartori, G. Barbanti Brodano, C. Griffoni, L. Martini, S. Boriani, M. Fini

**Affiliations:** ^1^Laboratory of Preclinical and Surgical Studies, Rizzoli Orthopedic Institute, Bologna, Italy; ^2^Laboratory of Biocompatibility, Technological Innovations and Advanced Therapies, Rizzoli Research Innovation Technology Department, Rizzoli Orthopedic Institute, Bologna, Italy; ^3^Department of Oncological and Degenerative Spine Surgery, Rizzoli Orthopedic Institute, Bologna, Italy

## Abstract

The use of spinal fusion procedures has rapidly augmented over the last decades and although autogenous bone graft is the “gold standard” for these procedures, alternatives to its use have been investigated over many years. A number of emerging strategies as well as tissue engineering with mesenchymal stem cells (MSCs) have been planned to enhance spinal fusion rate. This descriptive systematic literature review summarizes the in vivo studies, dealing with the use of MSCs in spinal arthrodesis surgery and the state of the art in clinical applications. The review has yielded promising evidence supporting the use of MSCs as a cell-based therapy in spinal fusion procedures, thus representing a suitable biological approach able to reduce the high cost of osteoinductive factors as well as the high dose needed to induce bone formation. Nevertheless, despite the fact that MSCs therapy is an interesting and important opportunity of research, in this review it was detected that there are still doubts about the optimal cell concentration and delivery method as well as the ideal implantation techniques and the type of scaffolds for cell delivery. Thus, further inquiry is necessary to carefully evaluate the clinical safety and efficacy of MSCs use in spine fusion.

## 1. Introduction

Spinal fusion is a common means to treat vertebral instability. Its use has quickly increased over the last decades in order to realize the stabilization of the spine in patients affected by degenerative, oncologic, and traumatic spine diseases. Autogenous bone harvested from the iliac crests is the standard procedure for spinal fusion surgery and it is used in more than 190.000 cases/year in Europe [1]. It owns all the key graft material properties, that is to say osteoconduction, osteoinduction, osteogenic potential, and also structural integrity if corticals are comprised. However, the use of autologous bone graft has been described to be linked with 5% to 35% nonunion rate, intraoperative blood loss, and residual morbidity at the donor sites in about 30% of the patients [2]. There are many factors inherent to the spine fusion failure such as tensile forces, low bone surface, and interference by surrounding musculature [3]. In addition, the time required for spinal fusion increases with advancing age and the fusion rate remains unpredictable in the ageing population [4]. Moreover, smoking, osteoporosis, and systemic illnesses have an adverse impact on bone and in particular in spinal surgery [5, 6]. The presence of these intrinsic complications has given rise to research into new materials and methods avoiding iliac crest harvesting. Thus, there are differing lines of research such as bone substitutes (allografts, demineralized bone matrix, and ceramics) and osteoinductive growth factors (bone morphogenic proteins). However, bone substitutes, which are merely osteoconductive and not osteoinductive, remain yet to be finished as substitutes for bone because the fusion achieved with them is not solid enough. In fact, for a successful spinal fusion to occur, several essential elements in addition to a biocompatible scaffold are necessary. They include the presence of the bone-forming cells or their precursors and an appropriate biological signal that direct bone synthesis. The most critical of these components are the osteoblasts or their precursors, the mesenchymal stem cell (MSC), both of which own the ability to form bone. To overcome these limitations, researchers have focused on new treatments that will allow for safe and successful bone repair and regeneration. In this field, adult stem cells derived from mesenchymal tissue represent a promising source for bone engineering for their ability to differentiate into osteoblasts. MSCs are undifferentiated cells characterized by a high proliferation rate that were found in several adult tissues [7–9]. The multipotent nature of individual MSCs was first established in 1999 by Pittenger et al. [10], and since then they have been found to be pluripotent, giving rise to endoderm, ectoderm, and mesoderm cells [11]. Thus, MSCs are well suited to therapeutic applications also because they can be easily cultured and have high ex vivo expansive potential [12–15]. In the treatment of several musculoskeletal injuries, such as bone, articular cartilage, and other joint tissues, MSCs from bone marrow (BMSCs) are the most widely used cells, followed by MSCs from adipose tissue (ADSCs) [16–18]. Both types of cells have been demonstrated to have a significant effect on spinal fusion in a multitude of settings including a variety of culturing mechanisms, scaffolds, and added growth factors. However, MSCs represent a lesser (0.001–0.01%) fraction of the total population of the nucleated cells [19, 20]. To increase the concentration of MSCs, several techniques have been developed, especially cell ex vivo expansion, but many problems limited the clinical application, such as the sterility technique, long culture time, high cost, and the mixture of human cell culture medium with fetal bovine serum. Thus, the method of collecting MSCs, as well as the real number of MSCs to be transplanted, remains yet to be established.

To date, a great body of research on MSCs for spinal fusion procedures was performed in vitro and in vivo but a clinical customary procedure for the use of cell-based strategies for spinal fusion surgery has not been established and contrasting clinical but also preclinical results were reported in literature. More importantly, the clinical transferability of some protocols is still to be settled, to optimize time and sources when modified/stimulated cells, custom made scaffolds, and in vitro steps are required [21]. Thus, in this systematic review, we aimed to evaluate the efficacy of MSCs in spinal arthrodesis procedures considering the preclinical and clinical studies of the last 10 years to shed light on using MSCs for spinal fusion treatment.

## 2. Motivations

### 2.1. Why a Systematic Review?

We have seen the necessity for performing a descriptive systematic literature review on MSCs use in spinal arthrodesis procedures in order to understand if the use of MSCs may represent a valid strategy able to facilitate and accelerate bone regeneration during spine surgery providing to researchers and clinicians a beginning point with solid foundations allowing this field to make a leap forward. Our aim is to offer answers to questions such as the following: “Since bone contains a complex environment of many cell types, are MSCs able to perform all the necessary physiological functions to achieve, facilitate, and accelerate spinal fusion?,” “What happens to MSCs when they are added to a scaffold?,” “Which source of MSCs is better to use and which techniques (ex vivo expansion and one-step procedure) are better to use?,” “How much does the existing preclinical model reflect the data so far collected in clinical studies?,” and “What do we have to do to further clarify the potential role of MSCs in spinal fusion procedures?” Specifically, we want to summarize the knowledge collected in nearly 10 years of research, learning from previous preclinical and clinical research which used MSCs for spinal fusion procedures, since there is an exigent need to have successful spinal fusion.

## 3. Methods

### 3.1. Descriptive Systematic Literature Review

Our descriptive literature review involved a systematic search that was carried out, according to the Preferred Reporting Items for Systematic Reviews and Meta-Analyses (PRISMA) statement, in three databases (https://www.ncbi.nlm.nih.gov/pubmed, https://www.webofknowledge.com, and https://www.scopus.com). In order to evaluate the ongoing clinical studies, the https://www.clinicaltrials.gov website was also checked. The keywords were mesenchymal stromal cells OR mesenchymal stem cells OR mesenchymal/progenitor stromal cells OR mesenchymal/progenitor stem cells AND spinal arthrodesis OR spinal fusion OR interbody fusion OR vertebral arthrodesis OR vertebral fusion. We sought to identify studies where MSCs were employed for spinal fusion procedures. Publications from 2006 to 2016 (original articles in English) were included. A public reference manager (“http://www.mendeley.com”) was used to delete duplicate articles.

## 4. Results and Discussion

**Figure 1:**
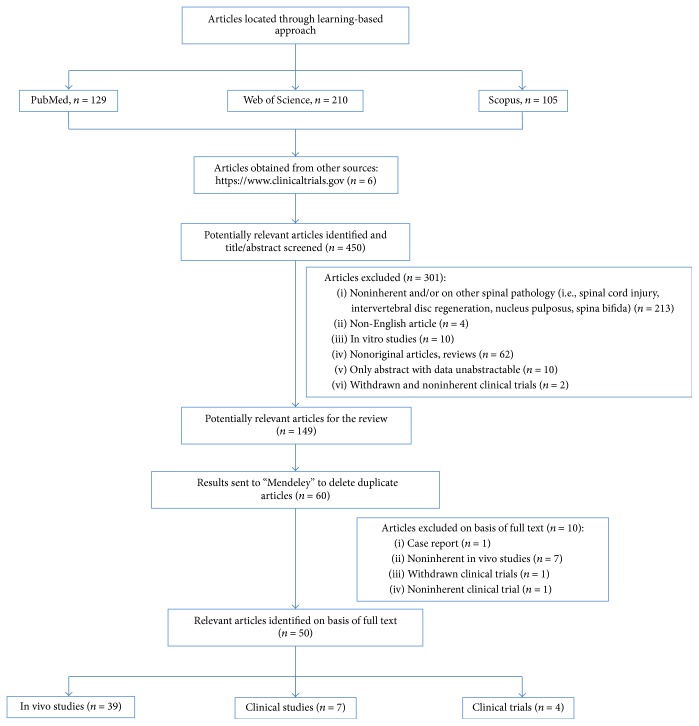
Systematic literature review flow diagram. Flow of information through the different phases of the systematic review.

**Figure 2:**
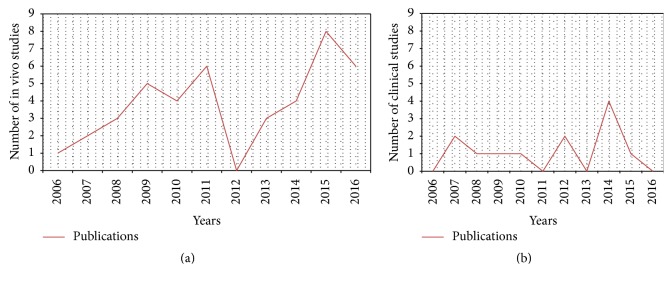
Historical distribution of (a) in vivo models and (b) clinical studies on MSCs use in spinal arthrodesis procedures according to the year of publication.

An initial literature search retrieved 444 references (Figure 1). Hundred and twenty-nine articles were identified using https://www.ncbi.nlm.nih.gov/pubmed, 210 articles were identified using https://www.webofknowledge.com, and 105 articles were found in https://www.scopus.com. Six additional articles were obtained from the website https://www.clinicaltrials.gov. The resulting references were selected for supplementary analysis based on the title and abstracts and 149 were considered eligible. References were submitted to a public reference manager (Mendeley 1.14, “https://www.mendeley.com”) to eliminate duplicate articles. Sixty complete articles were then reviewed to establish whether the publication met the inclusion criteria and 50 articles were recognized eligible for the review considering publications from 2006 to 2016 (Figures 2(a) and 2(b)). Thirty-nine articles were in vivo studies on small, medium, and large animal models (Tables 1, 2, and 3) while the remaining 11 articles were clinical studies or clinical trials (Tables 4 and 5).

**Figure 3:**
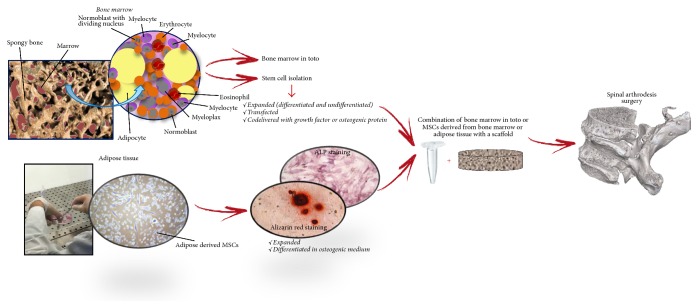
Flow chart summarizing the main steps of spinal fusion procedure when stem cell therapy is used.

Figure 3 summarizes the main steps of spinal fusion stem cell-based therapy founded in this literature search.

We did not perform meta-analyses of the selected studies but reported the results in a descriptive fashion. By considering the studies emerging from this review, we stratified the papers according to in vivo studies (small, medium, and large animal models) and clinical trials.

### 4.1. In Vivo Studies

#### 4.1.1. Small Animal Models

Thirteen studies (Table 1) employed MSCs in small animal models (*n* = 1 in mice and *n* = 12 in rats) in order to achieve or improve spinal fusion rate. In the majority of the studies the spinal fusion surgery was carried out by decortications of L4 and L5 but also L1-L2 [25] and L4–L6 [28] transverse processes. Klíma et al. [30] used titanium microplates and titanium screws to fix the spinous processes of L1–L3 vertebrae. The experimental time after surgery ranges from 4 to 8 weeks. Most of the studies used in vitro expanded MSCs [22, 25–27, 29–34] principally derived from bone marrow [25–27, 29, 30, 33, 34] but also from adipose tissue [22, 31–33]. Beyond the use of expanded MSCs, some authors, in order to take advantage not only by the mesenchymal component but also by the presence of trophic factors, cytokines, and extracellular matrix molecule, used bone marrow in toto [23, 24, 28]. Few studies used autologous MSCs [23, 29, 34] while the majority employed allogenic MSCs [22, 24–26, 30–33]. All the examined studies involved seeding cells into allograft [22, 27, 32] or into various scaffolds, such as ceramic [25, 30, 34], collagen sponge [24, 28, 31, 33], silk fibroin [26], and composites [23, 29]. Only one author employed autologous bone graft as control material [26] while the others used allografts or scaffolds without MSCs as control. When expanded MSCs were used the cell number, loaded on allografts or scaffolds, is 1.0–1.50 × 10^6^ [26, 29, 32–34] but also lower concentration as in the study of Lee et al. (0.25 × 10^6^) [22] or higher concentration was used [23, 26, 30, 31]. Differently from the studies where bone marrow in toto [23, 24, 28] and undifferentiated MSCs were cultured and loaded on a scaffold [25, 26, 30, 32–34] four studies [22, 29, 31, 34] employed cells cultured in osteogenic differentiation medium. In particular, Rao et al. examined also the role of low doses bone morphogenic protein- (BMP-) 2 codelivered with both undifferentiated and differentiated BMSCs showing that undifferentiated BMSCs with low-dose BMP-2 loaded on a composite scaffold demonstrated superior fusion rate in comparison to all the other examined groups. Low dose of BMP-2 was also evaluated in association with bone marrow in toto loaded on a collagen sponge, showing that fresh bone marrow aspirate increases the osteogenic potency and biologic efficiency of BMP-2 [24, 28] also in comparison to BMP-2 associated with other adjuvant factors such as platelet rich plasma [28]. BMP-2 was used also by Miyazaki et al. in an athymic rat model to compare the efficacy of human ADSCs and BMSCs transduced with an adenovirus containing the cDNA for BMP-2 loaded on collagen sponge. Authors showed that ADSCs transfected with adeno-BMP-2 induce abundant bone formation in a manner similar to genetically modified BMSCs [33]. Similar results were also obtained by Hsu et al. [31] that demonstrate the potential of adipose derived stem cell as cellular vehicle for this osteoinductive factor. Contrary to the positive effect on spinal fusion derived by the association of MSCs or bone marrow with BMP-2, the association of fibroblast growth factor-4 (FGF-4) with differentiated BMSCs loaded on a HA scaffold did not stimulate fusion but appears to induce fibrotic change rather than differentiation to bone [34]. Despite these negative results, recently Shih et al. [23] suggested a good performance in promoting spinal fusion rate associating new biomineralized matrices with bone marrow or basic FGF. Differently from the above-mentioned studies that used growth factors in association with BMSCs to enhance spinal fusion, other authors determined the efficacy of a *β*-tricalcium phosphate (TCP)/demineralized bone matrix (DBM) [22] or DBM alone [32] loaded with different doses of human perivascular stem cells (hPSCs) also in presence and absence of osteogenic protein NELL-1. Authors highlighted that both in healthy [32] and in osteoporotic condition [22] the presence of hPSCs [32] also in association with NELL-1 significantly improved spinal fusion [22]. Differently from all the other studies, Klíma et al. [30] adopted an instrumented model of interspinous fusion showing a nonsignificant new bone formation in animals treated with hydroxyapatite (HA) and BMSCs in comparison to animals treated with scaffold alone, even if in presence of BMSCs authors described minor inflammatory reaction compared to the animals treated without BMSCs. Finally, since the fate and contribution of the MSCs are not sufficiently clarified, especially at clinically relevant locations, Geuze et al. [25] using the bioluminescence imaging of luciferase-marked MSCs and adopting different experimental setup tried to elucidate and clarify the contribution made not only by MSCs itself on spinal fusion but also by the paracrine effect of MSCs when loaded on a ceramic scaffold. Results suggested that the soluble factors or the presence of extracellular matrix was not sufficient to induce bone formation; thus unfortunately they did not provide an answer to the critical question whether the principal mechanism of action of MSCs is based on their activity on the release of soluble mediators.

#### 4.1.2. Medium Animal Model

MSCs treatment to achieve spinal fusion was employed in 16 in vivo studies that used medium sized animal models (Table 2). All the studies used a single level posterolateral transverse process arthrodesis between L4-L5 or L5-L6 or L6-L7. With the exception of one study [44] spinal fusion surgery was carried out by creating a defect between L4 and L5 (depth of 5 mm and diameter of 10 mm); in all the others studies a transverse process decortication was performed. The experimental time after surgery ranges from 4 up to 18 weeks. Unless for the study by Koga et al. [36] that use fresh bone marrow to enhance the spinal fusion rate all the other authors used expanded, autologous [2, 35, 37–40, 42–47, 49], or allogeneic [41, 48] MSCs isolated from bone marrow [2, 35, 37, 38, 41–49] or adipose tissue [40] at different dosages (from 1.0 × 10^6^ cells to 1.0 × 10^8^). Some authors used MSCs with osteogenic differentiation [35, 37–40, 42, 44–47, 49] to increase the fusion rate while other used undifferentiated MSCs [2, 36, 41, 43, 48]. In all the studies MSCs were loaded on a scaffold (i.e., ceramic, polymeric, collagen sponge, and gelatin sponge) with the exception of Urrutia et al. [38] that used a pellet of cultured BMSCs cografted with an autologous bone graft and showed that adding differentiated BMSCs in a pellet without a scaffold not only failed to increase fusion rate, but completely inhibited bony growth. Differently, Nakajima et al. [35], using differentiated BMSCs plus HA, obtained a high rate of lumbar fusion similar to that obtained using autograft alone. In most of the studies the experimental treatment with MSCs was compared with autologous bone but in some of them this comparison is missing [2, 41–43, 45, 47, 48]. Niu et al. [42] compared BMSCs cultured in a biphasic calcium phosphate with BMSCs cultured with coralline HA. Coralline HA was used also by Chen et al. [49] who used MSCs fluorescent labeled with PKH-67 dye in combination with a bioresorbable hydrogel and coralline HA in comparison to autograft showing similar results between groups. In addition, to the use of a ceramic graft in association with BMSCs, several authors used BMP to enhance the osteogenic potency of MSCs [37, 43, 46] showing that MSCs in combination with BMP-2 enhanced bone formation in posterolateral spine fusion exerting a more osteoinductive action than MSCs alone [46]. Favorable results were also obtained comparing the association of a composite [43] and a ceramic [48] material with baculovirus genetically modified BMSCs overexpressing BMP-7 [43] or BMP-2 associated vascular endothelial growth factor (VEGF) [48] with nongenetically modified BMSCs. Additionally, Minamide et al. [37] tested also the hypothesis that both BMP-2 and basic fibroblast growth factor (FGF) mutually acted on the proliferation and osteogenic differentiation of rabbit BMSCs. They showed that the combined treatment with BMP-2 and basic FGF produced a favorable degree of spinal fusion comparable to autograft. An increased spinal fusion rate was also obtained by Koga et al. [36] assessing the osteogenic potential of HA sticks soaked with fresh bone marrow and fibronectin (FN). Interesting were also the results obtained by Hui et al. [2] that underlined that the combination of synthetic biomaterials, autologous differentiated BMSCs, and also low-intensity pulsed ultrasound promote spinal fusion. Differently from the use of low-intensity pulsed ultrasound, the use of hyperbaric oxygen therapy administrated to the animals did not enhance the spinal fusion rate when a combination of allogenic differentiate MSCs/alginate scaffold was evaluated [47]. The effectiveness of autologous differentiated BMSCs was evaluated also by Yang et al. [44] in association with a collagen sponge showing a high fusion rate similar to autologous bone. Another approach exploited by Douglas et al. is the ex vivo transfer of a gene encoding an osteoinductive factor to BMSCs which are subsequently reimplanted into the host. In this study Smad1C gene was transferred into rabbit MSCs isolated from bone marrow. The rationale for the use of this approach is to control more efficiently bone formation mimicking the natural cascade signals and avoiding the drawbacks associated with the direct use of BMPs. Authors showed that animals BMSCs transduced ex vivo with the Smad1C-expressing tropism-modified Ad5 vector mediated a greater amount of new bone formation than BMSCs transduced with any other vector [45]. Differently from all the other studies Urrutia et al. [38] evaluating a composite of hot compression-molded PLGA, HA, and type I collagen as an BMSCs carrier for a posterolateral spinal fusion used also a PKH fluorescence labeling system and highlighted that the transplanted BMSCs were partly responsible for the new bone formation. Positive results were also obtained using allogeneic undifferentiated rabbit BMSCs added to a type I collagen and calcium phosphate ceramics that promote spinal fusion and did not induce an adverse immune response [41]. Only one study evaluated the effectiveness of autologous ADSCs combined with a new mineralized collagen matrix (nHAC–PLA) for posterolateral spinal fusion. Results indicated that the rate of fusion was significantly higher in the autologous bone and ADSCs + nHAC-PLA groups than that in the nHAC-PLA and autologous bone + nHAC-PLA groups, demonstrating the effective impact of the scaffold also when combined with ADSCs [40].

#### 4.1.3. Large Animal Models

Ten studies used ovine as large animal models (Table 3) [51–57, 59] while two used swine [50, 58]. These studies carried out four-level, three-level, two-level, or single level spinal fusions surgery all instrumented with screws and bars with the exception of Gupta et al. that used a single level noninstrumented lumbar fusion [59]. In these studies both autologous bone marrow or expanded MSCs [50, 51, 54, 55, 58] and allogeneic bone marrow or mesenchymal precursor cells [52, 53, 56, 57, 59] were used to enhance spinal fusion and all of them involved seeding cells into allografts [50], collagen scaffolds [55], ceramics [51–54, 56, 57, 59], and composites [58] scaffolds. Except for Schubert et al. who use MSCs derived from adipose tissue [50] and Goldschlager et al. that used amnion epithelial cells [57] all the other authors used differentiated MSCs and mesenchymal precursor cells derived from bone marrow but also bone marrow in toto loaded on the scaffolds at different dosages. Wheeler et al. also compared different dosages of MSCs [52, 53, 56]. All the researchers used autografts and grafts without cells as controls and the majority highlighted superior result of the graft associated with MSCs and bone marrow in comparison to the graft alone, while similar [50] or best [51–53, 55, 56, 59] results were seen for autografts in comparison to grafts associated with MSCs. In addition, Goldschlager et al. showed superior results of mesenchymal precursor cells loaded on* Mastergraft* material also in comparison to amnion epithelial cells [57]. Differently from the above-mentioned studies, Cuenca-López et al. observed that bone autografts performed better than MSCs loaded on hybrid constructs [54]. Inferior results were also observed for MSCs loaded on a composite scaffold in comparison to autograft but also in comparison to scaffold associated with BMP-2 [58].

### 4.2. Clinical Studies

The search strategy identified 10 clinical studies (Table 4) about MSCs used for spinal fusion procedures. Among these 10 articles, three were excluded: for two articles we found only the abstract and not the full-text and the other one was a case report of an 88-year-old multidiseased osteoporotic patient treated with corticocancellous bone allograft, augmented with autologous bone marrow concentrate from iliac crest aspirate enriched with platelet rich fibrin from peripheral blood. Thus, due to the treatment protocol and the lack of a control group, the real contribution of MSCs on spinal fusion procedure could not be extrapolated. Therefore, 7 articles were analyzed: by comparing the characteristics of each study, it is evident that, in all studies, with the exception of the study by Moro-Barrero et al. [60], the authors employed the concentrate autologous bone marrow in comparison to fresh one inside the operating theater [20, 61–65]. Three studies associated bone marrow with a ceramic graft [20, 60, 61] while the remaining 4 combined bone marrow with allograft [62–65]. Three studies were prospective, randomized trials [61–63], two of which with limited number of patients [61, 62], while one was a prospective, multicenter, nonrandomized study on 182 patients [65]. All the studies withdrew the bone marrow from iliac crest and performed spinal fusion surgery on 1, 2, or 3 levels with similar surgical procedures and approaches. As far as the number of transplanted cells, cell concentration was not always reported as cell number in one milliliter or was not reported at all, thus making comparison among studies extremely difficult. In addition, another variable among studies is the method used for cell concentration (cell separator based on the density gradient centrifugation, centrifugation over a gradient, or without any gradient). Obviously, the absence of procedural and methodological guidelines affected the cell yield and thus it was not possible to identify a range for the cells number to be transplanted and to correlate it with the clinical outcome. In addition to the cells number, in two studies a control group was not used [64, 65], while other two studies compared the experimental treatment with autologous bone [60, 62] and one study with allograft chips alone [63]. Another study by Gan et al. compared autologous enriched MSCs/*β*-TCP with locally harvested bone combined with autologous enriched MSCs/*β*-TCP [20], while Odri et al. in a simple blind randomized clinical, prospective, monocentric study compared a biphasic calcium phosphate ceramics graft that was associated with autologous bone and concentrated bone marrow with unconcentrated bone marrow with ceramics graft and autologous bone [61]. The follow-up of the analyzed studies ranged from 12 months to 36.5 months [20, 60–65], demonstrating, through radiographic and clinical analyses, the safety and in one case a greater efficacy [63] of MSCs use in spinal fusion; however, these studies were conducted in too small patient cohorts and there is the need to confirm these data also from preclinical animal models, where transplanted cell phenotype, fate, and contribution to healing could be monitored and quantitatively measured to exclude malignant transformation.

As of August 2016, the ongoing clinical trials on MSCs for spinal fusion applications found through https://www.clinicaltrials.gov web site are 6 (Table 5). One of them was excluded because the objective of the trial was the definition of the osteogenic potential of MSCs and their progenitors during spinal fusion complication (pseudarthrosis). Another trial has not been analyzed because it has been withdrawn prior to the patients enrollment. The remaining 4 trials were all interventional study of phase I-II with a minimum follow-up of 12 months. Of them 2 were completed. In detail the trials assessed (1) the feasibility and safety of ex vivo expanded autologous MSCs fixed in allogenic bone tissue in comparison to autologous bone; (2) the effectiveness of autologous mesenchymal stem cells arranged in a calcium phosphate ceramic; (3) the effectiveness of allograft alone versus allograft with bone marrow concentrate; (4) the feasibility, safety, and tolerability of 3 different doses of immunoselected, culture-expanded, nucleated, allogenic mesenchymal precursor cells combined with resorbable ceramic granules in comparison to autograft alone. In all studies fusion surgery, surgical procedures, clinical approaches, and follow-up evaluations were similar. However, each trial was different from the other for patients number, MSCs manipulation or strategy, study arms, and presence and/or type of control group. In addition, the information available was not always complete. In some cases it was not clear which strategy would be employed for MSC manipulation and almost all the studies did not indicate the number of cells or the medium for cell infusion. Thus, although some studies could provide useful information, it was evident that more controlled clinical trials are necessary to understand whether MSCs can be successfully employed in spinal fusion procedures.

## 5. Conclusion and Future Prospective

In recent years, the basic and preclinical research literature clearly indicates the use of MSCs also in combination with various scaffolds, to repair bone defects, and many studies concern their use also for the treatment of vertebral instability. Thus, with the rapidly growing number of spine fusion surgeries performed annually, we have seen the need for performing this descriptive systematic literature review on MSCs use in spinal arthrodesis procedures in order to elucidate if the use of MSCs may really represent a valid strategy able to facilitate and accelerate spinal fusion.

In this review, several therapeutic strategies for the enhancement of spinal fusion rate based on stem cells have been developed in both preclinical and clinical studies. The application of an allograft or a scaffold, prevalently ceramics, associated with stem cells was adopted in all preclinical studies while the application of autograft, but also ceramic scaffolds, still in association with stem cell was used in the clinical setting (tissue engineering strategy). However, the use of growth factors (principally BMP-2) and other osteoinductive factors, as well as ex vivo gene therapy, was taken into consideration.

We found that numerous preliminary researches in this review were carried out in small, medium, and large animal models showing the potential for MSCs use in spinal fusion procedures. Despite the fact that in some of these studies adipose derived mesenchymal stem cells, human perivascular stem cells, and also amnion epithelial cells were used, the majority of the studies employed bone marrow cells. Based on these preclinical data it would seem that MSCs are able to perform the necessary physiological functions to achieve, facilitate, and accelerate spinal fusion. However, none of these examined studies was able to give a detailed elucidation about the fate of MSCs when they were added to a scaffold, although the success demonstrated that, in the animal models, some barriers remain prior to this therapy translation into the clinical setting. In fact, this review underlines that there are few and basic clinical trials, although some of them have shown that bone marrow cells used in humans can give a successful spine fusion. Some critical existing limitations include also the choice of the optimal cell concentration, the delivery method, the ideal manipulation procedure (ex vivo expansion and one-step procedure), and the best implantation techniques. In addition, researches that examine the optimal MSCs concentration are needed in large animal model, which are more similar to humans. These critical points also highlight the need for methods able to maximize the number of MSCs collected, as well as the presence of easy and feasible techniques in the clinical scenario. However, other matters that need further consideration comprise also the elimination of fetal calf serum, the possible reversibility of the differentiated state, the survival of the cells in vivo, the integration with the preexisting bone, and the capacity to form bone and marrow in vivo.

In conclusion, the use of MSCs as a cell-based therapy may represent a biological approach to reduce the high cost of osteoinductive factors as well as the high dose needed to induce bone formation. Thus, implementing this available potential treatment based on MSCs use and probably mitigating some adverse effects would make this kind of approach a possible therapeutic tool. Finally, although MSCs therapy remains an interesting and important opportunity of research, it is necessary that the spine surgery community carefully evaluates the safety and efficacy of MSCs use in spine fusion through randomized controlled and blinded clinical trials.

## Figures and Tables

**Table 1:** Published in vivo studies in small animal models on mesenchymal stem cells for spinal arthrodesis procedures.

Animal model	MSCs source	Other biological adjuvant	Scaffold material	Experimental time (weeks)	Spinal fusion level	Experimental design	Main outcome	Reference
Ovariectomized rat	hPSCs from adipose tissue of patients with and without osteoporosis	NELL-1	DBM/*β*-TCP	4 weeks	L4-L5	*Group 1*: DBM/*β*-TCP with hPSCs (0.25 × 10^6^ cells/mL) *Group 2*: DBM/*β*-TCP with hPSCs (0.75 × 10^6^ cells/mL) *Group 3*: DBM/*β*-TCP with NELL-1 (33.3 *μ*g/mL) *Group 4*: DBM/*β*-TCP with NELL-1 (66.6 *μ*g/mL) *Group 5*: DBM/*β*-TCP with hPSCs/NELL-1 at the dosage of groups 1 and 3 *Group 6*: DBM/*β*-TCP with a hPSCs/NELL-1 at the dosage of groups 2 and 4	(i) Group 1 achieved a fusion rate of 20% (1/5), group 2 of 28.6% (2/7), groups 3 and 4 of 20% (1/5), and group 5 of 37.5% (3/8), and group 6 improved the fusion rates up to approximately 83.3% (5/6) (ii) Microcomputed tomography imaging and quantification further confirmed solid bony fusion in group 6	[22]

Rat	In toto rat bone marrow from femur flush (1.1 × 10^7^ cells/mL)	bFGF	PEGDA-co-A6ACA hydrogels (poly(ethylene glycol)-diacrylate hydrogel (PEGDA) and N-acryloyl 6-aminocaproic acid (A6ACA))	2, 4, 6, and 8 weeks	L4-L5	*Group 1*: scaffold with bone marrow *Group2*: scaffold with bFGF *Group 3*: scaffold with saline solution	(i) Radiographs showed fusion masses in 4 animals out of 7 in each group at 2 weeks. At 4 weeks, all animals showed clear evidence of hard tissue formation, with progressively increase at 6 and 8 weeks (ii) *µ*-CT imaging at 8 weeks revealed a 51% of mineralized hard tissue for group 3, 59% for group 2, and 54% for group 1 (iii) Manual palpation provided evidence of fusion in all groups, with no significant differences in fusion indices	[23]

Rat	Fresh bone marrow (BM) cells (range, 0.60 to 2.60 × 10^6^ BM cells)	rhBMP-2 (0.006 mg/mL)	Absorbable collagen sponge (ACS)	8 weeks	L4-L5	*Group 1*: 2ACS with fresh BM and rhBMP-2 *Group 2*: 2ACS with rhBMP-2 *Group 3*: 1ACS with rhBMP-2 *Group 4*: ACS with BM *Group 5*: ACS alone	(i) In group 1 BM plus rhBMP-2/ACS significantly increased the fusion rate to 89% (16/18) compared with a base fusion rate of 33% (4/12) in group 3 and 50% (6/12) in group 2 (*p* < 0.05) (ii) No difference in strength or stiffness was detected among group 1 and groups 2 and 3. (iii) No fusion or bone formation was observed in the rats of groups 4 and 5	[24]

Rat	Expanded MSCs (3 × 10^6^) from goat BM iliac crest lentivirally transduced to express luciferase	None	HA/*β*-TCP	7 weeks	L1-L2 and L4-L5	*Group 1*: no cells *Group 2*: MSCs *Group 3*: MSCs gamma-irradiated (30 Gy) *Group 4*: MSCs dipped in liquid N_2_	(i) The antiluciferase immunohistochemistry showed no newly formed bone or luciferase-positive cells. (ii) Histological staining with Hematoxylin/Eosin highlighted no signs of a bone formation in any groups	[25]

Rat	Expanded bone marrow from rat femur (1 × 10^7^ cell/mL)	None	Silk fibroin (SF) and mineralized silk fibroin (mSF)	12 weeks	L4-L5	*Group 1*: SF scaffold *Group 2*: SF with MSCs *Group 3*: mSF *Group 4*: mSF with MSCs *Group 5*: autograft *Group 6*: sham group	Fusion rate, bone volume, biomechanical parameters, and histological score showed no significant differences between group 4 and group 5. Group 3 was significantly greater for most parameters than group 2	[26]

Rat	Allogenic MSCs	None		8 weeks	L4-L5	*Group 1*: trinity evolution (DBM with MSCs) *Group 2*: grafton (DBM) *Group 3*: DBM *Group 4*: decortication only	(i) Fusion rate by radiography was 8/8 for group 1, 3/8 for group 2, and 5/8 for group 3 (ii) Fusion rate by *µ*-CT and manual palpation was 4/8 for group 1, 3/8 for group 2, and 3/8 for group 3	[27]

Mouse	Bone marrow from femur and tibia (1.0 × 10^8^ cells/mL)	PRP from donor (1.0 × 10^9^ platelets/mL) or rhBMP-2 (31 *µ*g/mL)	ACS	4 weeks	L4-L5 and L5-L6	*Group 1*: collagen sponge with rhBMP-2 and saline solution *Group 2*: collagen sponge with rhBMP-2 and PRP *Group 3*: collagen sponge with rhBMP-2 and BM *Group 4*: decortication only	(i) Fusion appeared radiographically and histologically similar in all three experimental groups (ii) The area, volume, and density of the fusion mass were significantly greater (*p* < 0.05) for group 3 as compared with group 1 (iii) Group 2 had intermediate fusion area and density (iv) No spinal fusion was detected in group 4	[28]

Rat	Expanded rat bone marrow from femurs (1 × 10^6^ cells/mL)	Fibrin matrix	PCL-TCP	6 weeks	L4-L5	*Group 1*: 10 *µ*g of rhBMP-2 with 1 × 10^6^ undifferentiated BMSCs *Group 2*: 10 *µ*g of rhBMP-2 with osteogenic-differentiated BMSCs *Group 3*: 2.5 *µ*g rhBMP-2 with undifferentiated BMSCs *Group 4*: 2.5 *µ*g rhBMP-2 with osteogenic-differentiated BMSCs *Group 5*: 0.5 *µ*g rhBMP-2 with undifferentiated BMSCs *Group 6*: 0.5 *µ*g rhBMP-2 with osteogenic differentiated BMSCs	(i) Predifferentiation of BMSCs before transplantation failed to promote posterolateral spinal fusion when codelivered with low-dose of rhBMP-2 in group 5 as 17% fusion rate was observed (1/6) (ii) In contrast, combined delivery of undifferentiated BMSCs with low-dose BMP-2 (2.5 *µ*g) as in group 5 demonstrated significantly higher fusion rate (4/6 or 67%) as well as significantly increased volume of new bone formation	[29]

Rat	Human bone marrow (5 × 10^6^ MSCs)	None	Titanium microplates with HA	8 weeks	L1–L3	*Group 1*: titanium microplates with HA *Group 2*: titanium microplates with HA/MSCs	Histology, histomorphometry, and *µ*-CT revealed no significant bone formation in group 2 in comparison with group 1	[30]

Rat	ADSCs (5 × 10^6^ cells/scaffold)	rhBMP-2 or adenoviral vector containing BMP-2 gene	Type-I collagen sponge	4 weeks	L4-L5	*Group 1*: ADSCs transduced with an adenoviral vector containing rhBMP-2 gene *Group 2*: ADSCs with osteogenic media and 1 mg/mL of recombinant rhBMP-2 *Group 3*: rhBMP-2 (10 mg) *Group 4*: rhBMP-2 (1 mg) *Group 5*: ADSCs	(i) All animals of group 1 were characterized by fusion masses (8/8) after 4 weeks (ii) Group 1 revealed spinal fusion at the cephalad level (L3 and L4) (iii) New bone formation in groups 1 was significantly larger than those in any other treatment group (*p* < 0.005) (iv) Groups 3 and 4 showed a solid fusion in 8/8 and 4/8 animals, respectively (v) Groups 2 and 5 showed no fusion	[31]

Rat	hPSCs from adipose tissue	None	DBM	4 weeks	L4-L5	*Group 1*: DBM *Group 2*: DBM with 0.15 × 10^6^ hPSCs *Group 3*: DBM with 0.50 × 10^6^ hPSCs *Group 4*: DBM with 1.50 × 10^6^ hPSCs	(i) hPSC treatment (groups 2, 3, and 4) significantly increased spinal fusion rates in comparison with group 1 (ii) Groups 2, 3, and 4 resulted in fusion rates of 100%, 80%, and 100%, respectively, compared with 20% fusion in group 1 (iii) Computerized biomechanical simulation (finite element analysis) further demonstrated bone fusion in hPSC treatment groups (iv) Histological analyses showed endochondral ossification in hPSC-treated samples	[32]

Rat	ADSCs from healthy donors (1.0 × 10^6^) Purchased BMSCs (1.0 × 10^6^)	Adenoviral vectors adeno-*BMP-2 *and adeno-*LacZ* used to transduce ADSCs and BMSCs	ACS	8 weeks	L4-L5	*Group 1* ACS with ADSCs transfected with adeno-BMP-2 *Group 2* ACS with BMSCs transfected with adeno-BMP-2 *Group 3* ACS with rhBMP-2 *Group 4* ACS with ADSCs transfected with adeno-LacZ *Group 5* ACS with BMSCs transfected with adeno-LacZ, and *Group 6* ACS	(i) Spinal fusion was observed in groups 1, 2, and 3 rats (ii) 75% (15/20) of the animals of groups I and II had spontaneous extension of the fusion to a second level (iii) No animals in groups 4, 5, and 6 rats developed fusion (iv) New bone volume was significantly greater in groups 1 and 2 than in group 4	[33]

Rat	Expanded BM cells from femurs and tibias (1 × 10^6^/60 *μ*L)	FGF-4 (41 *μ*g)	HA	8 weeks	L4-L5	*Group 1*: HA *Group 2*: HA with MSCs *Group 3*: HA with MSCs and FGF-4	(i) Radiographic, high-resolution *μ*-CT, and manual palpation revealed spinal fusion in 5/6 (83%) in group 2 (ii) In group 1, 3/6 (60%) rats developed fusion at L4-L5 by radiography and 2/5 (40%) by manual palpation in radiographic examination (iii) In group 3, bone fusion was observed in only 50% of rats by manual palpation and radiographic examination	[34]

**Table 2:** Published in vivo studies in medium animal models on mesenchymal stem cells for spinal arthrodesis procedures.

Animal model	MSCs source	Other biological adjuvant	Scaffold material	Experimental time (weeks)	Spinal fusion level	Experimental design	Main outcome	Reference
Rabbit	Expanded BM from iliac crest (1.5 × 10^6^ cells/mL)	Osteogenic medium	HA	6 weeks	L5-L6	*Group 1*: autograft *Group 2*: HA with type I collagen gel *Group 3*: HA and type I collagen gel with MSCs *Group 4*: HA and type I collagen gel with MSCs induced toward osteogenic phenotype	The fusion rates were 4/6 in group 1; 0/6 in group 2; 2/6 in group 3; and 4/5 in group 4	[35]

Rabbit	Fresh BM from iliac crests	Fibronectin	HA	6 weeks	L4-L5	*Group 1*: autograft from iliac crest *Group 2*: autograft from transverse process bone graft *Group 3*: HA sticks and iliac bone graft *Group 4*: HA sticks with BM aspirate *Group 5*: HA sticks *Group 6*: HA sticks with FN and BM aspirate. *Group 7 ***: **decortication only	(i) The elasticity and mechanical strength were significantly higher in group 1 than in groups 2, 4, and 5 (ii) The mechanical strength achieved in groups 3 and 6 was nearly equal to that in group 1 (iii) The mechanical strength was significantly higher in group 6 than in group 4 (iv) Histology showed intraporous osteogenesis in groups 3, 4, and 6	[36]

Rabbit	Expanded BM cells from iliac crest (1 × 10^6^cells/mL)	(i) rhBMP-2 (ii) bFGF (iii) Autograft	HA	6 weeks	L4–L5	*Group 1*: autograft *Group 2*: HA with MSCs *Group 3*: HA with MSCs and BMP *Group 4*: HA with MSCs and bFGF *Group 5*: HA with MSCs and BMP/bFGF	The fusion rates were 4/7 in autograft group; 0/7 in MSCs/HA group; 2/7 in MSCs/HA/BMP group; 3/7 in MSCs/HA/FGF group; and 6/7 in MSCs/HA/BMP/bFGF group	[37]

Rabbit	Expanded BM cells from iliac crest	None	None	8 weeks	L4-L5	*Group 1*: autograft *Group 2*: autograft with MSCs	(i) In group 1, the fusion rate was 53% (8/15) (ii) In group 2, the fusion rate was 0%	[38]

Rabbit	BM from femur, tibia, trochanter, and iliac crest	None	TCP	7 weeks	L5-L6	*Group 1*: TCP alone *Group 2*: TCP with MSCs *Group 3*: TCP with MSCs and LIPUS	(i) Significant increase in manual palpation in group 3 treated with LIPUS (86%) in comparison with groups 1 (0%) and 2 (14%) without LIPUS (ii) The bone volume of fusion mass was significantly larger in group 3 than the other two groups by quantitative computed tomographic analysis (iii) Group 3 fusion mass had a better osteointegration length between host bone and implanted composite and presented more new bone formed in the TCP implants (iv) Group 3 had osteochondral bridging, early stage of bony fusion, from histological point of view	[2]

Rabbit	Expanded BM from iliac crest	None	Poly(lactide-co-glycolide) (PLGA)/HA/ type I collagen	6 weeks or 12 weeks after grafting	L4-L5	*Group 1*: autograft *Group 2*: PLGA/HA/Type I collagen with MSCs	Radiographic, computed tomography examinations, torsional loading tests, and histologic examinations showed solid fusion in 3/5 rabbits in both experimental groups at 6 weeks and 5/5 solid fusion in both groups at 12 weeks	[39]

Rabbit	ADSCs from the inguinal groove	None	Nano-hydroxyapatite–collagen–polylactic acid (nHAC–PLA)	10 weeks	L5-L6	*Group 1*: autograft *Group 2*: nHAC–PLA *Group 3*: autograft with nHAC–PLA *Group 4*: ADSCs with nHAC–PLA	(i) The rate of fusion was significantly higher in group 1 and group 4 than in group 2 and group 3 (ii) Microstructural analysis of the samples showed more new bone-like tissue formation in group 1 and group 4 than in the other two groups (iii) Mechanical properties showed that the strength and stiffness of group 1 and group 4 were much higher than those of group 2 and group 3	[40]

Rabbit	BM from femur (1.0 × 10^8^ allogeneic MSCs)	None	Bioresorbable purified fibrillar collagen and calcium phosphate ceramics containing HA and *β*- TCP	18 weeks	L5-L6	*Group 1*: HA/ *β*-TCP with MSCs *Group 2*: HA/ *β*-TCP	(i) In group 1 CT scanning revealed excellent fusion in 2/12 rabbits (17%), good fusion in 8/12 (66%), and fair fusion in 2/12 (17%) (ii) In group 2 a good fusion result was found in 3/12 rabbits (25%), fair fusion in 6/12 (50%), and poor fusion in 3/12 (25%)	[41]

Rabbit	Expanded human BM from iliac crest (10^7^)	None	PLGA/BCP/collagen graft and MSC/PLGA/coralline HA/collagen graft	10 weeks	L4-L5	PLGA/BCP/collagen with MSCs (on the left side) PLGA/coralline HA/collagen with MSCs (on the right side)	(i) Radiographic, CT, and bone mineral content analyses showed continuous bone bridges and fusion mass incorporated with the transverse processes (ii) Bone mineral content values were higher in MSCs/PLGA/BCP/collagen group than in MSCs/PLGA/coralline HA/collagen group	[42]

Rabbit	Expanded BM from iliac crest (2 × 10^7^)	Bac-BMP-7	Collagen/TCP/HA	12 weeks	L4-L5	*Group 1*: collagen/TCP/HA *Group 2*: collagen/TCP/HA with MSCs *Group 3*: collagen/TCP/HA/ Bac-BMP-7 with MSCs	(i) In the CT results, 6/12 fused segments were observed in group 1 (50%), 8/12 in group 2 (67%), and 12/12 in group 3 (100%) (ii) The fusion rate by manual palpation was 0% (0/6) in group 1, 0% (0/6) in group 2, and 83% (5/6) in group 3 (iii) Histology showed that group 3 had more new bone and matured marrow formation	[43]

Rabbit	Expanded and osteogenic induced BM from iliac crest (OMSCs)	None	ACS	8 and 12 weeks	L4-L5	*Group 1*: ACS with OMSCs *Group 2*: ACS *Group 3*: autograft *Group 4*: nothing	(i) Bony fusion was evident as early as 8 weeks in groups 1 and 3 (ii) At 8 and 12 weeks, by CT and histologic analysis, new bone formation was observed in groups 1 and 3 and fibrous tissue and absence of new bone were present in groups 2 and 4 (iii) Manual palpation showed bony fusion in 40% (4/10) of rabbits in group 1, 70% (7/10) of rabbits in group 3, and 0% (0/10) of rabbits in both groups 2 and 4	[44]

Rabbit	Expanded BM from iliac crest (10^5^)	MSCs transduced with Smad1C gene	Absorbable gelatin sponge	4 weeks	L6-L7	*Group 1*: BMSCs transduced with Smad1c with Ad5 vector *Group 2*: BMSCs transduced with Smad1c with Ad5 vector retargeted to *α*_*v*_ integrins (RGD) *Group 3*: BMSCs transduced with BMP-2 with Ad5 vector *Group 4*: BMSCs transduced with BMP-2 with Ad5 vector retargeted to *α*_*v*_ integrins (RGD) *Group 5*: BMSCs transduced with an Ad5 vector expressing b-galactosidase	(i) The area of new bone formed in groups 1, 2, 3, and 4 was significantly greater than the area of new bone formed in group 5 (*p *< 0.04 for each group compared with group 5) (ii) Group 4 mediated a greater amount of new bone formation than group 3 (iii) Similarly, group 2 mediated a greater amount of new bone formation than group 1 (*p* < 0.0007) (iv) Group 2 mediated a greater amount of new bone formation than the other groups (*p* < 0.02)	[45]

Rabbit	Expanded and osteogenic induced BM from iliac crest (2 × 10^6^)	rhBMP-2	Alginate scaffold	16 weeks	L4-L5	*Group 1*: autograft *Group 2*: alginate scaffold with MSCs *Group 3*: alginate scaffold with MSCs and rhBMP-2 *Group 4*: alginate scaffold with rhBMP-2	(i) Radiographic union of group 1 was 11/12, of group 2 8/11, of group 3 11/12, and of group 4 0/12 (ii) Manual palpation highlighted 6/6 solid fusion in group 1, 1/6 in group 2, 5/6 in group 3, and 0/6 in group 4 (iii) The mechanical analysis (failure torque) did not differ significantly between group 1 and group 3 that were both higher than group 2	[46]

Rabbit	Expanded and osteogenic induced BM from iliac crest (2 × 10^6^)	None	Alginate scaffold	12 weeks	L4-L5	*Group 1*: alginate scaffold *Group 2*: alginate scaffold with MSCs *Group 3*: alginate scaffold/ hyperbaric oxygen (HBO) therapy with MSCs	Radiographic examination and manual palpation highlighted no union for group 1 (0/12), 10/22 for group 2, and 6/12 for group 3	[47]

Rabbit	Expanded BM from iliac crest	TCP	Recombinant baculovirus encoding BMP-2 (Bac-CB) and vascular endothelial growth factor (Bac-VEGF)	12 weeks	L4-L5	*Group 1*: TCP *Group 2*: TCP with MSC *Group 3*: TCP with MSCs/Bac	(i) Radiographically fusion rate was detected as being 0/12 in group 1, 4/12 in group 2, and 10/12 in group 3 (ii) Manual palpation highlighted no fusions in group 1, two solid fusions in group 2, and five solid fusions in group 3	[48]

Rabbit	Expanded and osteogenic induced BM from iliac crest	Bioresorbable hydrogel (pluronic F27) and coralline HA	None	6 and 12 weeks	L4-L5	*Group 1*: Pluronic 127/HA hybrid graft with MSCs *Group 2*: autograft	(i) Solid fusion was achieved in 3/5 rabbits from both group 1 and 2 at 6 weeks, and solid fusion was present in 5/5 from both group at 12 weeks (ii) No differences were detected between the two groups for biomechanical analysis and from histological point of view	[49]

**Table 3:** Published in vivo studies in large animal models on mesenchymal stem cells for spinal arthrodesis procedures.

Animal model	MSCs source	Other biological adjuvant	Scaffold material	Experimental time (weeks)	Spinal fusion level	Experimental design	Main outcome	Reference
Pig	ADSCs from inguinal subcutaneous tissue	None	DBM	8 and 12 weeks	L2–L6	*Group 1*: one cage was left and three filled with freeze dried irradiated cancellous pig bone graft *Group 2*: freeze dried irradiated cancellous pig bone graft *Group 3*: cancellous bone autograft *Group 4*: bone graft with 3D osteogenic differentiated ADSCs	*µ*-CT scan, microradiography, and histology/histomorphometry demonstrated a significant increase in bone content in group 4	[50]

Sheep	Expanded and osteogenic induced BMSCs from iliac crest (5-6 × 10^7^)	Fibrin	TCP/HA	12 weeks	L1–L6	*Group 1*: HA with MSCs *Group 2*: TCP/HA with MSCs *Group 3*: autograft	(i) Radiography, manual palpation, histological analysis, and SEM analyses revealed demonstrated better bone formation in group 2 compared to group 1 (ii) Histomorphometry detected 55.8% of new bone in group 3, followed by group 2 (42.7%) and group 1 (10.7%)	[51]

Sheep	Allogenic sheep mesenchymal precursor cells (MPCs) from BM from iliac crest	None	HA/TCP	16–36 weeks	L2–L5	*Group 1*: autograft *Group 2*: HA/TCP *Group 3*: HA/TCP with MPCs (25 × 10^6^) *Group 4*: HA/TCP with MPCs (75 × 10^6^) *Group 5*: HA/TCP with MPCs (225 × 10^6^)	Computed tomography, high-resolution radiography, biomechanical testing, organ pathology, bone histopathology, and bone histomorphometry showed that allogeneic mesenchymal precursor cells produced fusion efficacy similar to that achieved using iliac crest autograft	[52]

Sheep	Allogenic MPCs from BM from sheep iliac crest	None	HA/TCP	16 weeks	L4-L5	*Group 1*: autograft *Group 2*: HA/TCP with MPCs (2.5 × 10^6^) *Group 3*: HA/TCP with MPCs (6.5 × 10^6^) *Group 4*: HA/TCP with MPCs (12.5 × 10^6^)	(i) Manual palpation of the fusion site indicated solid fusion in more than 75% of MPC-treated group and 65% of group 1 (ii) Computed tomography and histomorphometry analyses showed all animals in the MPCs groups and group 1 fusion masses were present at 16 weeks	[53]

Sheep	Expanded and osteoinduced BM from iliac crest	None	HA	6 months	L4-L5	*Group 1*: autograft *Group 2*: allograft *Group 3*: HA *Group 4*: HA with MSCs.	(i) By CT scan and histology lumbar fusion were higher for groups 1 and 2 (70%) than for group 3 (22%) and group 4 (35%) (ii) New bone formation was higher for groups 1 and 2 (iii) Group 4 had a better fusion rate than group 3, but the histology showed no significant differences between them in terms of quantity of bone formation	[54]

Sheep	BM concentrate (1.5 × 10^6^ in 0.2 mL)	None	Natural bone collagen scaffold (NBCS) from human organic bone particles	6 and 10 weeks	L3-L4 and L4-L5	*Group 1*: autograft *Group 2*: NBCS *Group 3*: BMCs *Group 4*: NBCS with BMCs	(i) Solid spinal fusion was achieved in all six segments (6/6) in group 4 at 10 weeks, compared with 4/8 segments in group 1, 2/8 segments in group 2, and 3/6 segments in group 3 (ii) The biomechanical stiffness of fusion masses and bone volume at the fusion site were higher in group 4 (*p* < 0.05) (iii) At 10 weeks, the radiographic score reached was significantly higher in group 4 than in groups 1, 2 and 3 (iv) Histological findings revealed that group 4 induced new bone formation integrated well with host bone tissue	[55]

Ewes	Allogenic MPCs (5 × 10^6^) or allogenic amnion epithelial stem cells (5 × 10^6^ AECs)	None	Fidji interbody cage made from polyetheretherketone and HA/TCP	3 months	C3-C4	*Group 1*: cage packed with autograft *Group 2*: cage packed with HA/TCP *Group 3*: cage packed with HA/TCP and MPCs *Group 4*: cage packed with HA/TCP and AECs *Group 5*: controls	(i) Significant fusion mass was detected in group 3 compared to that in groups 1, 2, or 4 (ii) CT scan at 3 months revealed that 5/6 animals in group 3 (83%) had continuous bony bridging compared with 0/ 5 of group 4 and 1/6 of group 1 and 2/6 of group 2 (*p* < 0.01)	[56]

Ewes	Allogeneic MPCs (5 × 10^6^ or 10 × 10^6^)	None	Fidji interbody cage made from polyetheretherketone and HA/TCP	3 months	C3-C4 anterior cervical discectomy and fusion with a interbody cage	*Group 1*: cage packed with autograft *Group 2*: cage packed with HA/TCP *Group 3*: cage packed with HA/TCP and 5 × 10^6^ MPCs *Group 4*: cage packed with HA/TCP and 10 × 10^6^ MPCs *Group 5*: controls	(i) No significant differences were found between groups 3 and 4 (ii) CT scan showed that 9/12 (75%) MPC-treated animals had continuous bony bridging compared with 1/6 of group 1 and 2/6 of group 2 (*p* < 0.019 and *p* < 0.044, resp.) (iii) By quantitative CT, density of new bone in MPC-treated animals was 121% higher than in group 2 (*p* < 0.017) and 128% higher than in group 1 (*p* < 0.0001)	[57]

Pig	BMSCs (10 × 10^6^)	rhBMP-2 (0.6 mg)	Bioresorbable scaffolds made from medical grade poly (Σ-caprolactone)-20% tricalcium phosphate (mPCL/TCP)	9 months	L2-L3 and L4-L5	*Group 1*: mPCL/TCP with rhBMP-2 *Group 2*: mPCL/TCP with BMSCs *Group 3*: mPCL/TCP *Group 4*: autograft	(i) The mean radiographic scores were 3.0, 1.7, 1.0, and 1.8 for groups 1 to 4, respectively (ii) The bone volume fraction of group 1 was twofold higher than group 2 (iii) Histology, *µ*-CT, and biomechanical evaluation showed solid and comparable fusion between groups 1 and 4 (iv) Group 2 showed inferior quality of fusion when compared with groups 1 and 4 while group 3 showed no fusion even at 9 months	[58]

Ovine	Autogenous whole BM or BM concentrate	None	TCP	6 months	L4-L5	*Group 1*: autograft *Group 2*: TCP with BM concentrate *Group 3*: TCP with whole bone marrow/ *Group 4*: TCP .	(i) At 6 months, 33% of group 2 and 25% of the group 1 sites were fused, compared with 8% of group 3 and 0% of group 4 (ii) Histology of fused samples showed denser bone formation in group 2 than in group 1 sites	[59]

**Table 4:** Published clinical studies involving the use of mesenchymal stem cells for spinal arthrodesis procedures.

Arthrodesis level	MSCs source	Cell manipulation	Treatment	Patient's number (mean age)	Follow-up	Complications	Reference
Single level = 22 2 or more levels = 13	Right posterosuperior iliac crest	Fresh bone marrow	(i) Left side: autologous bone graft (ii) Right side: mixture of BCP and fresh autogenous bone marrow	35 (24 males, 11 females) Mean age = 59.2	Minimum 30 months	1 pseudoarthrosis	[60]

Single level = 14 2 levels = 23 3 levels = 4	Right and left iliac crest	Bone marrow concentrate (enriched using a cell separator)	(i) Decompression cases: locally harvested bone combined with autologous enriched MSCs/*β*-TCP (ii) Nondecompression cases: autologous enriched MSCs/*β*-TCP	41 (30 men, 11 women) Mean age = 44.0	Median 36.5 months	(i) 4 patients with transient exudation or moderate swelling in their wounds (ii) 2 pseudoarthrosis (iii) 1 patient with bursa synovialis (iv) 1 patient with progressive instability of the adjoined supra-vertebra	[20]

1 and 2 levels	Posterior iliac crest	Bone marrow concentrate	(i) Side 1: concentrated bone marrow associated with macroporous biphasic calcium phosphate ceramics graft and autologous bone (ii) Side 2: nonconcentrated bone marrow with ceramics graft and autologous bone	15 Mean age = 46.3	24 months	None	[61]

1, 2, or 3 levels	One iliac crest	Bone marrow concentrated	(i) Side 1: allograft plus autologous bone marrow concentrate (ii) Side 2: autologous iliac crest bone	25 (15 males and 10 females) Mean age = 45.6	24 months	None	[62]

Not specified	Posterior iliac crests	Bone marrow concentrate	(i) 40 patients: allograft chips alone (ii) 40 patients: spongious allograft chips mixed with bone marrow concentrate	80 (22 men, 58 females)	24 months	Two complications occurred in each of the two groups: hematoma with subsequent revision surgery and drainage during the first week postoperatively	[63]

Not specified	Single iliac crest	Bone marrow concentrate	31 patients: concentrated bone marrow aspirate with allograft and demineralized bone matrix	31 (9 men and 22 females) Mean age: 71.5	At least 12 months	(i) One seroma (ii) One pseudarthrosis (iii) Three reoperation for 3 patients for adjacent segment pathology	[64]

1 or 2 levels	Non applicable	Allograft cellular bone matrix containing native mesenchymal stem cells and osteoprogenitor cells	182 patients: allograft cellular bone matrix containing native mesenchymal stem cells and osteoprogenitor cells	182 (49% female, 51% male) Mean age: 51	24 months	(i) 1 durotomy (ii) 2 wound infections (iii) 2 incidences of new radiculopathy (iv) 1 incidence of hypotension (v) 1 incidence of hypertension (vi) 2 incidences of postoperative soft-tissue swelling	[65]

**Table 5:** List of clinical trials involving mesenchymal stem cells for spinal arthrodesis procedures (from clinicaltrials.gov).

ClinicalTrials.gov Identifier	Condition	Study type	Estimated enrollment/ enrolled patients	MSC data (source, manipulation, or strategy)	Number of cells	Study arms	Follow-up (months)	Activity
NCT01552707	Degenerative spondylolisthesis grades I-II	Interventional phases 1-2	62	Expanded autologous mesenchymal stem cells obtained under GMP conditions fixed in allogenic bone tissue	Not reported	(i) Group 1: instrumented spinal fusion and the tissue engineering product composed by “ex vivo” expanded autologous mesenchymal stem cells fixed in allogenic bone tissue in spinal fusion (ii) Group 2: standard treatment of instrumented spinal fusion and patient's bone iliac crest	12 months	Recruiting

NCT00549913	Posterolateral lumbar fusion	Interventional phases 1-2	42	Immunoselected, culture-expanded, nucleated, allogeneic mesenchymal progenitor cells	Not reported	(i) Experimental group 1: lowest dose of NeoFuse (ii) Experimental group 2: middle dose of NeoFuse (iii) Experimental group 3: highest dose of NeoFuse (MPCs) (iv) Control group: autologous bone graft	24 and 36 months	Completed

NCT01513694	Intervertebral disc disease	Interventional phases 1-2	15	Cell suspension of MSCs from bone marrow aspirate expanded in vitro in a specific medium enriched with platelet lysate without addition of animal products	Not reported	(i) Autologous mesenchymal stem cells arranged in a phosphate ceramic	Not reported	Unknown

NCT01603836	Spondyloarthrosis, spondylosis	Interventional	80	Spongious allograft chips mixed with bone marrow concentrate	74 × 10^4^/L at average (range, 1.06–1.98 × 10^4^/L)	(i) Group 1: spongious allograft chips alone (ii) Group 2: spongious allograft chips mixed with bone marrow concentrate	24 months	Completed
